# Glioblastoma-derived Leptin Induces Tube Formation and Growth of Endothelial Cells: Comparison with VEGF Effects

**DOI:** 10.1186/1471-2407-11-303

**Published:** 2011-07-19

**Authors:** Rita Ferla, Maria Bonomi, Laszlo Otvos, Eva Surmacz

**Affiliations:** 1 Sbarro Institute for Cancer Research and Molecular Medicine, Biotechnology Center, Temple University, 1900 N 12th street, Philadelphia, PA 19122, USA; 2 Department of Medical Oncology, Policlinico G.B. Rossi, Piazzale L.A. Scuro 10, 37134 Verona, Italy; 3 Department of Biology, College of Science and Technology, Temple University, 1900 N 12th Street, Philadelphia, PA 19122, USA

## Abstract

**Background:**

Leptin is a pleiotropic hormone whose mitogenic and angiogenic activity has been implicated in the development and progression of several malignancies, including brain tumors. In human brain cancer, especially in glioblastoma multiforme (GBM), leptin and its receptor (ObR) are overexpressed relative to normal tissue. Until present, the potential of intratumoral leptin to exert proangiogenic effects on endothelial cells has not been addressed. Using *in vitro *models, we investigated if GBM can express leptin, if leptin can affect angiogenic and mitogenic potential of endothelial cells, and if its action can be inhibited with specific ObR antagonists. Leptin effects were compared with that induced by the best-characterized angiogenic regulator, VEGF.

**Results:**

We found that GBM cell lines LN18 and LN229 express leptin mRNA and LN18 cells secrete detectable amounts of leptin protein. Both lines also expressed and secreted VEGF. The conditioned medium (CM) of LN18 and LN 229 cultures as well as 200 ng/mL pure leptin or 50 ng/mL pure VEGF stimulated proliferation of human umbilical vein endothelial cells (HUVEC) at 24 h of treatment. Mitogenic effects of CM were ~2-fold greater than that of pure growth factors. Furthermore, CM treatment of HUVEC for 24 h increased tube formation by ~5.5-fold, while leptin increased tube formation by ~ 80% and VEGF by ~60% at 8 h. The mitogenic and angiogenic effects of both CM were blocked by Aca 1, a peptide ObR antagonist, and by SU1498, which inhibits the VEGF receptor. The best anti-angiogenic and cytostatic effects of Aca1 were obtained with 10 nM and 25 nM, respectively, while for SU1498, the best growth and angiogenic inhibition was observed at 5 μM. The combination of 5 μM SU1498 and Aca1 at 25 nM (growth inhibition) or at 10 nM (reduction of tube formation) produced superior effects compared with single agent treatments.

**Conclusions:**

Our data provide the first evidence that LN18 and LN 229 human GBM cells express leptin mRNA and might produce biologically active leptin, which can stimulate tube formation and enhance proliferation of endothelial cells. Furthermore, we demonstrate for the first time that a peptide ObR antagonist inhibits proangiogenic and growth effects of leptin on endothelial cells, and that the pharmacological potential of this compound might be combined with drugs targeting the VEGF pathway.

## Background

Leptin is an adipocyte-derived hormone that plays a major role in the regulation of body weight by inhibiting food intake and stimulating energy expenditure via hypothalamic-mediated effects [1,2]. Besides its anorexigenic function, leptin regulates several physiological processes, including angiogenesis [3-5]. Human endothelium and primary cultures of human endothelial cells express the leptin receptor, ObR [6,7]. *In vitro *studies demonstrated that leptin can stimulate growth and survival of endothelial cells as well as induce their migration and organization into capillary-like tubes [6-9]. *In vivo*, leptin is able to induce complete angiogenesis in the chick choriallantoic membrane assay [6] and disc angiogenesis system [10] as well as promote neovascularization in corneas of normal, but not ObR-deficient Zucker fa/fa, rats [7] or normal mice [11]. In addition to its own effects, leptin synergizes with vascular endothelial growth factor (VEGF) and basic fibroblastic growth factor (bFGF) in the stimulation of blood vessel growth and vascular permeability [11].

Proangiogenic and mitogenic functions of leptin have been implicated in development and progression of different neoplasms. Multiple studies demonstrated that leptin is able to stimulate survival [12-14], proliferation [15-17], migration and invasiveness [18-22] of several cancer cell types. In addition, leptin might also contribute to tumor neoangiogenesis. Exposure of cancer cells to hypoxic conditions and/or elevated concentrations of growth factors, such as insulin, can activate production of endogenous leptin, raising intratumoral levels of this hormone [23-28]. Proangiogenic effects of leptin can be further potentiated by its ability to upregulate the expression of other angiogenic factors, such as VEGF, bFGF, interleukin 1-β, and leukemia inhibitory factor in cancer cells [18,29-31].

New evidence suggests leptin can be involved in the development of brain tumors [13,22,32-35]. Initial work documented the presence of leptin and ObR transcripts in various human intracranial tumors [34]. Other reports demonstrated that rat glioma tissues and cell lines express leptin mRNA [33,36], and that in rat C6 cells leptin can increase survival [13,32,33] and enhance migration and invasion of these cells [22].

We recently demonstrated that both leptin and ObR proteins are overexpressed in human brain tumors relative to normal brain tissue, and that leptin/ObR expression levels positively correlate with the degree of malignancy. The highest levels of leptin and ObR were found in glioblastoma multiforme (GBM), where both proteins were coexpressed with activated forms of serine/threonine protein kinase B (Akt) and signal transducer and activator of transcription 3 (STAT3). Interestingly, the greatest amounts of all these proteins were detected in perivascular areas and in groups of cells invading the adjacent brain parenchyma [35].

In ObR-positive glioblastoma cell lines LN18 and LN229, leptin stimulates cell proliferation and induces STAT3 and Akt pathways as well as inactivates the cell cycle suppressor Rb [35]. Furthermore, leptin-dependent phosphorylation of STAT3 in LN18 and LN229 cells can be inhibited with Aca1, a novel ObR antagonist [37].

Until present, no studies addressed the potential angiogenic role of leptin in human GBM. Considering that glioma progression from lower-grade tumors to highly malignant GBM is characterized by increasing intratumoral expression of leptin [35] as well as induction of angiogenesis [38,39], we investigated angiogenic properties (induction of tube formation) of GBM-derived leptin using endothelial cell models and specific ObR antagonists. The effects were compared with that produced by VEGF, the best characterized angiogenic factor.

## Results

### Conditioned media of GBM cultures stimulate tube formation and growth of human vascular endothelial cells

The survival and expansion of brain tumor cells is associated with increased expression and secretion of proangiogenic factors [38-40]. New vessel formation requires that endothelial cells migrate into the extracellular matrix and then adhere to each other to create a lumen [38,41]. To examine the effect of GBM cell line-derived conditioned media (CM) on this process, we employed an *in vitro *model of angiogenesis using human umbilical vein endothelial cells (HUVEC). HUVEC have the ability to invade a collagen I matrix and to form a network of tube-like structures [9,41,42].

We first tested if conditioned media (CM) derived from our GBM cell lines can induce proliferation and tube formation of HUVEC. HUVEC were cultured for 24 h on collagen I in presence of CM from LN18 and LN229 cells mixed 1:1 with HUVEC growth medium. The ability of HUVEC to organize into tube-like structures was scored as the number of enclosed spaces (ES).

**Figure 1:**
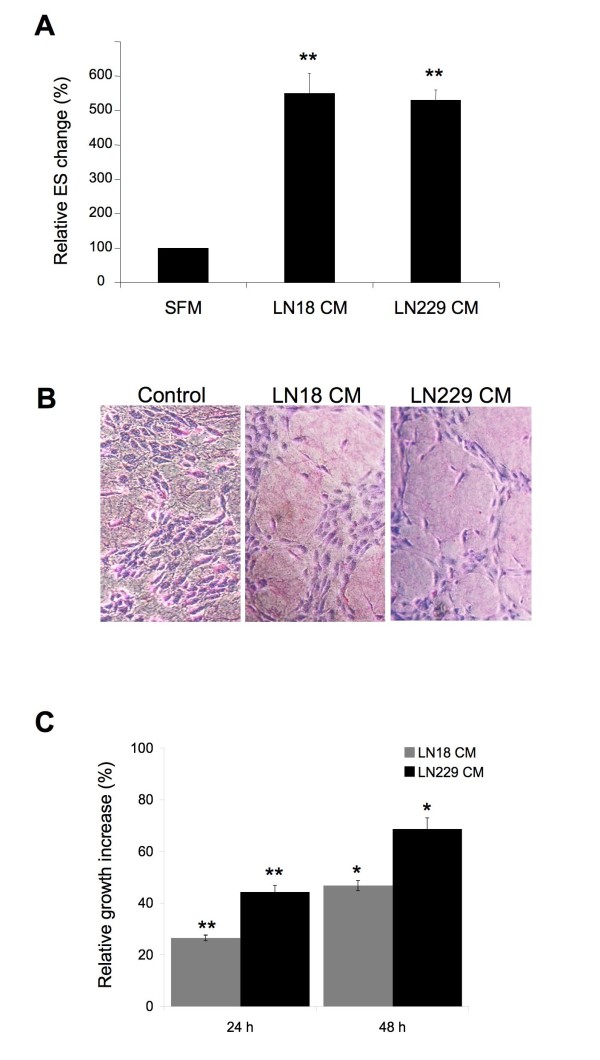
**CM derived from GBM cell lines LN18 and LN229 enhances angiogenic and mitogenic capabilities of HUVEC**. **A) **Tube-like formation assay was performed in SFM or LN18 and LN229-derived CM mixed (1:1) with VCBM for 24 h, as described in Materials & Methods. The graph shows relative % increase of ES formed by HUVEC with the number in SFM taken as = 100%. Columns, mean of at least three independent experiments done in triplicates; bars, SE. ** P < 0,001 (vs. control SFM: VCBM). **B) **Representative phase contrast pictures (magnification 10×) of HUVEC *in vitro *angiogenesis in response to LN18 or LN229 CM for 24 h. SFM:VCBM treatment was used as control. The cultures were evaluated and photographed as described in Materials and Methods. **C) **HUVEC were grown in SFM:VCBM or LN18 and LN229-derived CM mixed (1:1) with VCBM for 24 or 48 h. The graph represents relative growth increase with SFM:VCBM taken as reference (0%). Columns, mean of at least three independent experiments done in triplicates; bars, SE. * P < 0,05 and ** P < 0,001 (vs. control SFM:VCBM).

Incubation with LN18- and LN229-derived CM increased the number of ES by 5.7- and 5.3-fold, respectively, relative to negative control (Figure 1A). Moreover, relevant morphological changes in endothelial cells were noted. In response to treatment with both CM, endothelial cells become elongated, exhibited extended protrusions, and were aligned along the perimeter of the enclosed spaces. In contrast, in the negative control experiment, only a minimal invasion and formation of ES was noticeable (Figure 1B).

Endothelial cell proliferation is another key characteristic of the angiogenic process. A 24 or 48 h treatment with GBM-derived CM significantly increased the growth of HUVEC. In particular, LN18 and LN229-derived CM enhanced cell proliferation by ~ 26% and ~ 44% at 24 h, and ~ 47% and ~ 69% at 48 h, respectively (Figure 1C). All the above data suggest that LN18 and LN229 CM contain factors able to induce *in vitro *endothelial cell proliferation and differentiation.

### Analysis of leptin and VEGF mRNA and protein expression in LN18 and LN229 cells

**Figure 2:**
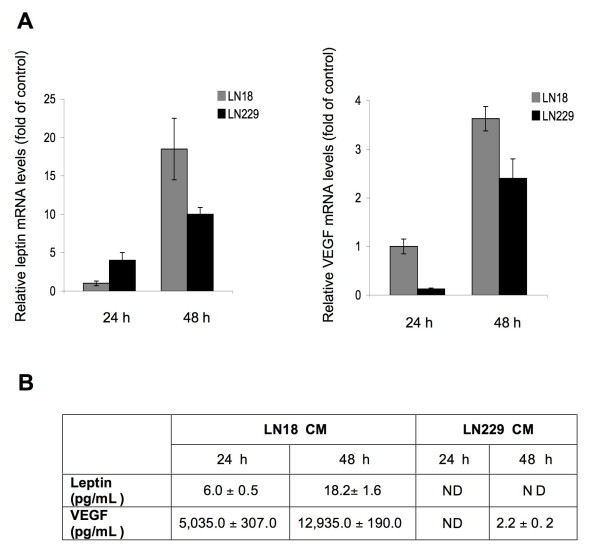
**GBM cells express leptin and VEGF**. **A) **The abundance of leptin and VEGF mRNA was studied with qRT-PCR as described in Materials and Methods. The graphs represent leptin and VEGF mRNA levels relative to LN18 levels in SFM (= 1) ± SD. **B) **Leptin and VEGF secreted protein levels were measured in LN18 and LN229 CM by ELISA, as described in Materials and Methods. The values represent pg/mL per 9 × 10^6 ^LN18 cells and 6 × 10^6 ^LN229 cells ± SD.

The expression of leptin mRNA and protein by human breast and colorectal cancer cells and rat glioblastoma cultures has been documented previously [24,27,33,43]. The synthesis of VEGF by GBM and other cancer cells has also been described [44-46]. Here we studied if LN18 and LN229 cell lines express leptin and VEGF mRNAs and proteins (Figure 2).

Leptin and VEGF mRNAs were detected in both cell lines, however, a cell-specific dynamic of expression was noted for both transcripts. At basal conditions, the levels of leptin mRNA were significantly lower (8.3-fold and 5.2-fold in LN18 and LN229 cells, respectively) than that of VEGF mRNA. In both cell lines, leptin mRNA levels were higher at 48 h than at 24 h in SFM. However, in LN229 cells, leptin mRNA levels at 24 h were ~5-fold greater than that in LN18 cells. On the other hand, after 48 h in SFM, leptin transcripts detected in LN229 cells were significantly lower than that in LN18 cells. Under our experimental conditions, LN18 cells showed an approximately 18-fold increase of leptin mRNA levels after 48 h of serum-starvation (Figure 2A). Less variability was observed for VEGF mRNA expression. VEGF mRNA levels increased in a time-dependent manner and were more elevated in LN18 cells than in LN229 cells at both time points (Figure 2A).

Next, we investigated the amounts of secreted leptin and VEGF in CM derived from both GBM cell lines (Figure 2B). At 24 h, we found ELISA-detectable levels of both leptin and VEGF only in LN18 cells, but not in LN229 cells. At 48 h, amounts of both proteins increased in LN18 CM, while in LN229 CM, leptin was undetectable and VEGF was present at very low levels (Figure 2B).

### Leptin and VEGF stimulate tube formation, growth and signaling in HUVEC. Inhibitors of ObR and VEGFR block these effects

**Figure 3:**
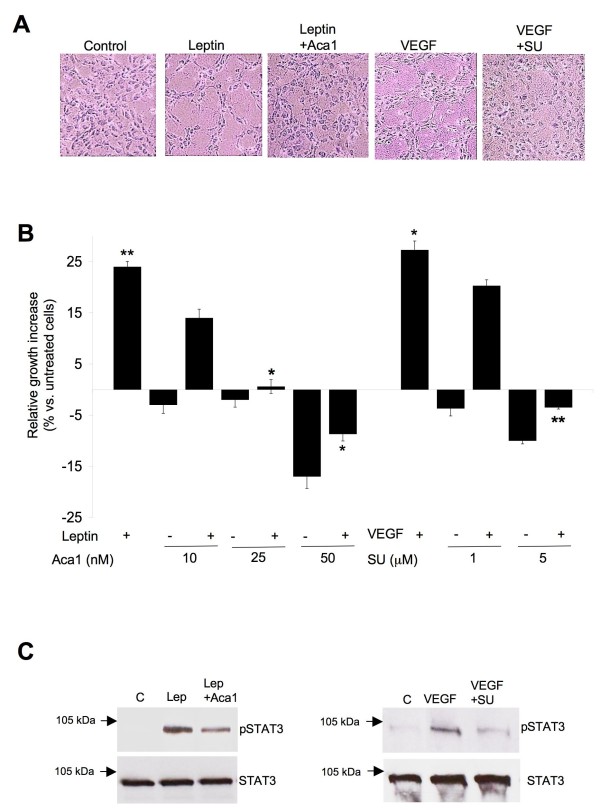
**Leptin and VEGF enhance angiogenesis and growth of HUVEC. ObR and VEGFR inhibitors counteract these effects**. **A) **Representative phase contrast images (magnification 10×) of ES formed by HUVEC treated with 100 ng/mL leptin and/or with Aca1 or with 50 ng/mL VEGF and/or with SU1498 or left untreated (control) for 8 h. **B) **HUVEC growing in VCBM were treated for 24 h with 200 ng/mL leptin and/or Aca1 10, 25 or 50 nM or with 50 ng/mL VEGF and/or or with SU1498 1 and 5 μM or left untreated (control). The graph represents relative growth increase with untreated cells taken as reference (0%). Columns, mean of at least three independent experiments done in triplicates; bars, SE. * P < 0,05 and ** P < 0,01 (Leptin or VEGF vs. untreated cells, leptin + Aca1 vs. leptin or VEGF + SU1498 vs. VEGF). **C) **HUVEC were pretreated for 1 h with ObR or VEGFR inhibitors and then treated with 200 ng/mL leptin or 50 ng/mL VEGF for 15 min or left untreated as described in Materials and Methods. Levels of pSTAT3 and total STAT3 (~88 kDa) were determined by WB in 50-70 μg of total cell lysates using specific Abs, as described in Material and Methods. Arrows indicate the position of 105 kDa molecular weight marker.

HUVEC are capable to respond to both leptin and VEGF, as they express various isoforms of ObR, including the long signaling form, ObRb, [6,7] as well as the VEGF receptor (VEGFR) [47,48]. As previously reported, leptin can stimulate tube-like structures *in vitro *[6-9]. To investigate the mechanism of this effect, we used Aca1, a potent ObR antagonist, developed in our laboratories and proven to inhibit leptin signaling in LN18 and LN229 cells [37]. Treatment of HUVEC with 100 ng/mL leptin for 8 h produced ~ 80% increase in ES formation compared with untreated cells (Table 1 and Figure 3A). Addition of Aca1 consistently counteracted this leptin-dependent effect. At the lowest concentration used (10 nM) Aca1 completely reverted the leptin-induced ES increase, whereas a slight reduction of the ES number vs. control was observed in the presence of Aca1 at 25 and 50 nM concentrations. Notably, Aca1 alone did not affect the number of ES relative to control, except for a slight decrease at the highest concentration, suggesting its specific activity towards ObR in presence of leptin (Table 1 and Figure 3A).

**Table 1:** Leptin and VEGF enhance ES formation by HUVEC.

Conditions	ES (%)
Untreated	100.0

Leptin	178.0 ± 3.0

Leptin + Aca1 10 nM	90.0 ± 1.0

Leptin + Aca1 25 nM	79.0 ± 6.5

Leptin + Aca1 50 nM	72.0 ± 32.5

Aca1 10 nM	103.5 ± 12.0

Aca1 25 nM	90.0 ± 1.0

Aca1 50 nM	68.0 ± 4.0

VEGF	160.0 ± 13.0

VEGF + SU1498 1 μM	121.0 ± 30.0

VEGF + SU1498 5 μM	98.0 ± 14.5

VEGF + SU1498 10 μM	73.0 ± 6.0

SU1498 1 μM	100.5 ± 23.0

SU1498 5 μM	91.0 ± 1.4

SU1498 10 μM	60.0 ± 10.0

ObR and VEGFR inhibitors counteract this effect.

Number of ES after treatment with 100 ng/mL leptin and/or Aca1Free 10, 25 and 50 nM or with 50 ng/mL VEGF and/or SU1498 1, 5 and 10 μM was scored as described in Materials and Methods. Untreated cells were taken as control (100%). Tube-like formation assay was performed for 8 h in VCBM. Means ± SD are shown.

In parallel, we treated HUVEC with 50 ng/mL VEGF, either alone or in presence of SU1498, a potent inhibitor of VEGFR2 [49]. VEGF increased by ~ 60% the number of ES, and this effect was antagonized by SU1498 in a dose-dependent manner, with the best response noted at 5 μM (Table 1 and Figure 3A).

Next, we assessed the proliferative response of HUVEC to leptin in the presence or absence of ObR antagonist. Leptin at 200 ng/mL increased the growth of HUVEC by 25% relative to control (untreated cells) (Figure 3B). The addition of Aca1 interfered with leptin-induced proliferation in a dose-dependent manner. In particular, Aca1 at 25 nM completely and significantly abolished leptin mitogenic effects (Figure 3B), while the antagonist at the highest concentration (50 nM) produced cytotoxic effects, significantly more pronounced in the absence of leptin. However, no great influence on cell growth was detected in HUVEC treated with Aca1 alone at 10 and 25 nM.

The parallel experiments with VEGF demonstrated that 50 ng/mL VEGF stimulated HUVEC proliferation by 27% relative to untreated cells. SU1498 reduced this effect in a dose dependent manner. 5 μM SU1498 totally blocked VEGF effects, while higher concentrations of the inhibitor were cytotoxic (Figure 3B).

To investigate the mechanism of Aca1 and SU1498 interference with leptin or VEGF effects on HUVEC, we studied if the antagonists are able to inhibit ligand-induced intracellular STAT3 signaling. The induction of STAT3 by leptin or VEGF in HUVEC was previously reported [7,50]. We confirmed that leptin activates STAT3 in these cells and found that Aca1 is able to significantly reduce leptin-dependent STAT3 phosphorylation (Figure 3C). Similarly, VEGF activated STAT3, and SU1498 reduced STAT3 phosphorylation in VEGF-treated HUVEC (Figure 3C).

These above data suggest that Aca1 and SU1498 are suitable to evaluate the specific contributions of leptin and VEGF in angiogenic and mitogenic effects of CM derived from GBM cell cultures.

### Effects of ObR and VEGFR inhibitors on CM-induced tube formation and growth of HUVEC

Our results demonstrated detectable amounts of leptin and VEGF mRNAs in LN18 CM, suggesting that these cells might produce leptin and VEGF proteins. In order to assess if the observed effects of LN18 CM on tube formation and growth of HUVEC can be ascribed to the activity of leptin and VEGF, we used Aca1 and SU1498, specific antagonists of ObR and VEGFR2, respectively.

**Figure 4:**
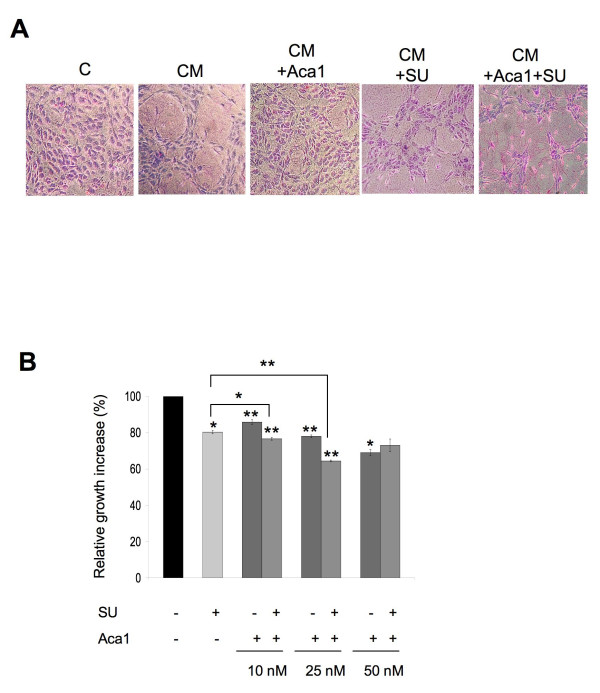
**Aca1 and SU1498 inhibit angiogenic and mitogenic effects of LN18 CM**. **A)** Representative phase contrast images (magnification 10×) of ES formed by HUVEC grown for 24 h in SFM or LN18-derived CM containing or not 50 nM Aca1 and/or 5 μM SU1498. **B)** HUVEC were grown for 48 h in CM mixed (1:1) with VCBM, containing 10, 25 or 50 nM Aca1 and/or 5 μM SU1498 as described in Materials and Methods. The graph represents relative growth increase, with CM taken as reference (100%). Columns, mean of at least three independent experiments done in triplicates; bars, SE. * P < 0,05 and ** P < 0,01 (CM + Aca1 or CM + SU1498 5 μM vs. CM alone, CM + Aca1/SU1498 5 μM vs. CM + Aca1 and CM + SU1498 5 μM).

The addition of Aca1 to LN18 CM significantly reduced the ability of HUVEC to reorganize into ES. Specifically, 10 nM and 25 nM Aca1 inhibited CM-dependent ES formation by 38 and 45%, respectively. This effect was not improved by increasing the concentration of Aca1 up to 50 nM (Table 2 and Figure 4A). Similarly, treatment with SU1498 blocked CM-induced ES formation by 45 and 75% at 1 and 5 μM, respectively (Table 2 and Figure 4A).

**Table 2:** Effects of Aca1 and SU1498 on LN18 CM-induced ES formation by HUVEC.

Conditions	ES (%)
LN18 CM	100.0

LN18 CM + Aca1 10 nM	62.0 ± 11.5

LN18 CM + Aca1 25 nM	55.0 ± 1.4

LN18 CM + Aca1 50 nM	52.0 ± 8.5

LN18 CM + SU1498 1 μM	55.0 ± 5.5

LN18 CM + Aca1 10 nM + SU1498 1 μM	35.0 ± 7.5

LN18 CM + SU1498 5 μM	25.0 ± 3.5

LN18 CM + Aca1 10 nM + SU1498 5 μM	10.2 ± 3.2

Tube-like formation assay was performed in LN18-derived CM mixed (1:1) with VCBM, containing or not Aca1 and/or SU1498 for 24 h, as described in Materials and Methods. The table shows the relative increase of ES formation (%), with LN18 CM taken as reference (100%). Means ± SD are shown.

The combination of the lowest effective dose of Aca1 with different doses of SU1498 produced greater ES inhibition than that seen with individual antagonists. Specifically, 10 nM Aca1 plus 1 μM SU1498 reduced ES formation by 65%, while 10 nM Aca1 with 5 μM SU1498 blocked ES organization by 90% (Table 2).

We also evaluated the effect of the antagonists on LN18 CM-dependent growth of HUVEC cultures (Figure 4B). Aca1 counteracted the effect on cell proliferation induced by LN18 CM in a dose-dependent manner. The greatest inhibition of growth was observed at 48 h when Aca1 at 10, 25, and 50 nM reduced the mitogenic effects of CM by 14, 22, and 31%, respectively (Figure 4B). SU1498 at 5 μM reduced LN18 CM-mediated growth of HUVEC by 20%, while no significant effect was observed with SU1498 1 μM and higher concentrations of the antagonists were slightly cytotoxic (Figure 4B and data not shown).

The combination of 25 nM Aca1 and 5 μM SU1498 reduced HUVEC proliferation by 45%, demonstrating the significant improvement over single inhibitor treatments. However, addition of Aca1 to 5 μM SU1498 only minimally increased cytostatic effects, while the combination of 50 nM Aca1 and 5 μ SU1498 did not improve the efficacy of single treatments (Figure 4B). These results suggested that LN18 CM affects, at least in part, HUVEC growth and tube formation through ObR and VEGFR2-dependent mechanisms, both of which can be targeted by specific molecular antagonists.

## Discussion

Malignant astrocytic gliomas, especially GBMs, are characterized by poor prognosis and low patient survival rates [51]. Although these tumors rarely metastasize, they almost always recur locally because of their inherent tendency for diffuse infiltration [52,53]. In particular, a strong induction of angiogenesis marks the transition from lower-grade tumors to more aggressive and lethal GBMs [39]. Therefore, despite advanced clinical approaches with surgery, radiotherapy and chemotherapy [54-56], inhibition of angiogenesis might represent a key strategy in the treatments of gliomas.

Recent preclinical data demonstrated that anti-VEGF agents (e.g. ceradinib, bevacizumab) can transiently normalize the elevated permeability and interstitial pressure of brain tumor vessels, enhancing in this way the penetration of concurrently administered drugs [38-40,52,57,58]. Besides direct VEGF or VEGFR2 inhibition for glioblastoma, clinical studies are being conducted or planned with agents targeting further downstream or alternative pathways frequently altered in brain tumors, including the mTOR/Akt and EGFR pathways [39].

Nevertheless, the success with the existing compounds in the management of brain tumors is very limited. It is likely that combination of therapeutic agents targeting different pathways, especially angiogenic pathways, will produce more significant clinical effects. In this context, we focused on leptin, a multifunctional hormone that is able to exert angiogenic activity in different *in vitro and in vivo *model systems [6-8,10,11,18,29-31].

Leptin has been implicated in neoplastic processes, especially in obesity-related cancers, where the hormone has been shown to stimulate cancer cells growth, survival [12,14,15,28,59], resistance to different chemotherapeutic agents [60,61] as well as migration, invasion and angiogenesis [18-21,29,62].

In the central nervous system (CNS) leptin regulates several physiological brain functions, including hippocampal and cortex-dependent learning, memory and cognitive function, neuronal stem cells maintenance, and neuronal and glial development [63,64]. In addition, recent research suggests the potential role of this hormone in the progression of brain tumors [35]. We previously demonstrated that the expression of leptin and ObR in human brain tumor tissues correlates with the degree of malignancy, and the highest levels of both markers are detected in GBM. Specifically, and in relevance to the present study, leptin and ObR were expressed in over 80% and 70% of 15 GBM tissues analyzed [35]. Other studies demonstrated leptin mRNA expression in rat glioma tissues and cell lines [33,36]. Because leptin and ObR in human brain tumors are commonly coexpressed, leptin effects are likely to be mediated by autocrine pathways. Using in vitro models, we found that LN18 and LN229 ObR-positive GBM cells respond to leptin with cell growth and induction of the oncogenic pathways of Akt and STAT3, as well as inactivation of the cell cycle suppressor Rb [35]. However, the potential role of intratumoral leptin in glioma progression, especially in the regulation of angiogenesis, has never been addressed. Here we investigated if the hormone can be expressed by human GBM cell cultures, if it can affect angiogenic and mitogenic potential of endothelial cells, and if its action can be inhibited with specific ObR antagonists. The results were compared with that induced by the best-characterized angiogenic regulator, VEGF.

Our data demonstrated that conditioned media produced by both LN18 and LN229 GBM cell lines enhanced HUVEC tube formation and proliferation. These data are in agreement with previous reports showing that GBM cultures express VEGF and other factors that can induce HUVEC angiogenesis [65-67].

We found variable levels of leptin and VEGF mRNA in LN18 and LN229 cell lines cultured under SFM conditions. In general, the abundance of VEGF transcripts in both cell lines was significantly greater that that of leptin mRNA. Secreted leptin and VEGF proteins were found in LN18 CM, while in LN229 CM, leptin was undetectable and VEGF was present at low levels. The reason for lack or minimal presence of these proteins in LN229 CM, despite quite prominent expression of the cognate mRNAs, is unclear. It is possible that it is due to limited sensitivity of ELISA assays unable to detect proteins below the minimal threshold level. We speculate that LN229 cells might produce proteins binding VEGF and leptin, thereby converting them into ELISA-unrecognizable complexes. Alternatively, LN229 CM might contain proteases degrading the angiogenic proteins.

In order to clarify if LN18 CM angiogenic and mitogenic effects are, at least in part, related to leptin secreted by these cells, we used specific ObR inhibitor, Aca1. We have previously demonstrated that this antagonist binds ObR in vitro, inhibits leptin-induced signaling at pM-low nM concentrations in different types of cancer cells, including LN18 and LN229 cells, while its derivative Allo-aca is able to reduce the growth of hormone-receptor positive breast cancer xenografts and enhance survival of animals bearing triple-negative breast cancer xenogranfts [37,68]. Furthermore, All-aca also inhibits leptin activity in some animal models of rheumatoid arthritis [69]. Interestingly, we also detected CNS activity of Aca1, suggesting that the peptide has the ability to pass the blood-brain barrier [37,68,70].

In the present work, we found that Aca 1 can abrogate leptin-induced tube formation and mitogenesis of HUVEC at 10 and 25 nM concentrations, respectively. Notably, the peptide alone did not affect cell growth and did not modulate the ability of HUVEC to organize into tube-like structures, suggesting that it acts as a competitive antagonist of ObR. Next, we demonstrated that Aca1 at 10-50 nM concentrations was able to antagonize tube formation and growth effects of LN18 CM. The anti-angiogenic effects of 25 and 50 nM Aca1 were comparable to that obtained with 1 μM SU1498, while anti-mitotic activity of 25 and 50 nM Aca1 was comparable to the action of 5 μM SU1498. Furthermore, the combination of low doses of Aca1 (10 nM) and SU1498 (1 or 5 μM) produced greater inhibition of CM effects than that obtained with single antagonists.

Interestingly, Aca1 or SU1498 appeared to differentially affect the morphology of HUVEC cultures. While Aca1 reverted the organized ES phenotype to the initial appearance of dispersed cell culture, SU1498 disrupted ES structures, reduced cell-matrix attachment and induced cell aggregation. This might suggest that the inhibitors affect different cellular mechanism and that leptin and VEGF control HUVEC biology through different pathways.

Taken together, our data indicated that GBM cells are able to induce endothelial cells proliferation and organization in capillary-like structures through, at least in part, leptin- and VEGF-dependent mechanisms. Thus, leptin might contribute to the progression of GBM through the stimulation of new vessel formation. Leptin action can be direct or indirect, through upregulation of VEGF expression. Indeed, we observed that leptin can transiently increase VEGF mRNA levels in GBM cells at 6-8 h of treatment (data not shown). In this context, effective reduction of tube formation and mitogenic activity of endothelial cells by ObR antagonist, especially in the combination with VEGFR2 inhibitor, suggest that targeting both leptin and VEGF pathways might represent a new therapeutic strategy to treat GBM.

## Conclusions

Our previous work demonstrated that leptin and ObR are significantly overexpressed in human GBM tissues and the presence of both biomarkers correlates with tumor grade. Current data suggest that human GBM cells in culture have the ability produce biologically active leptin that can induce growth and pro-angiogenic effects in endothelial cells. These effects of leptin can be blocked with a novel ObR antagonist, Aca1. The pharmacological potential of this compound might be combined with novel drugs targeting the VEGF pathway.

## Methods

### Cell lines and growth conditions

All cell lines were obtained from ATCC (Manassas, VA). Glioblastoma cell lines LN229 and LN18 were cultured in low glucose-Dulbecco modified Eagle's Medium (DMEM) (Cellgro Mediatech, Manassas, VA) containing 5% fetal bovine serum (Cellgro Mediatech, Manassas, VA). Human Umbilical Vein Endothelial Cells (HUVEC) were maintained in Vascular Cell Basal Medium (VCBM), supplemented with the Vascular Cell Growth kit BBE, both purchased from ATCC.

### ObR and VEGFR inhibitors

The ObR antagonist, Aca1, is a short leptin-based peptidomimetic (H-Thr-Glu-Nva-Val-Ala-Leu-Ser-Arg-Aca-NH2) whose sequence is based on leptin/ObR binding site III. The process of peptide design, screening and development has been reported by us before [37,71,72]. The efficacy of Aca1 and its derivative Allo-aca *in vitro *and *in vivo *has been described in detail previously [37,68]. SU1498, the antagonist of VEGFR2 was purchased from Calbiochem, USA.

### Conditioned medium (CM) preparation

Subconfluent LN18 and LN229 cell cultures were placed in SFM (DMEM low glucose supplemented with 0.42 g/mL bovine serum albumin, 1 μM FeSO_4 _and 2 mM L-glutamine) for 24 or 48 h, and then the CM was collected, centrifuged at 2000 rpm for 5 min, and the supernatants frozen at -80°C until use. The number of cells in cultures used for CM production was counted.

### Proliferation assays

HUVEC (1.5 × 10^4^) were plated in 24-well plates and allowed to adhere overnight in the growth medium. Then the cells were treated for 24 h with either 200 ng/mL leptin (R&D Systems, Minneapolis, MN) in presence or absence of 10, 25 or 50 nM Aca1, or with 50 ng/ml VEGF in presence or absence of 1 or 5 μM SU1498 or left untreated as control. For assays with GBM-derived CM, HUVEC were seeded as described above, and allowed to adhere overnight. Then the culture medium was replaced with SFM (negative control) or CM mixed with HUVEC growth medium (1:1) with or without Aca1 (10, 25, 50 nM) and/or SU1498 5 μM. At conclusion of proliferation assays, the cells were counted under the microscope with trypan-blue exclusion. Each experiment was performed in triplicate and repeated at least three times.

### *In vitro *tube formation assay

The tube formation assay was based on procedures described by Park et al and Feng et al. [9,42]. For the tube-like formation assays, 24-wells plates were coated with 300 μl of 2 mg/mL collagen I (BD Biosciences, Bedford, MA) prepared according to manufacturer's instructions. Where appropriate, leptin (100 ng/mL) and/or Aca1 (10, 25, 50 nM) and/or VEGF (50 ng/ml) and/or SU1498 (1, 5, 10 μM) were added to the collagen I prior to polymerization. Then, 8 × 10^4 ^of HUVEC suspended in 1 ml of HUVEC growth medium containing various treatments were plated on the top of the collagen layers. For tube formation assay performed with CM, HUVEC were seeded in 1 ml of SFM (negative control) or GBM-derived CM mixed (1:1) with HUVEC growth medium, containing or not Aca1 and/or SU1498. After 8 and 24 h for assays performed in HUVEC growth medium and CM, respectively, the HUVEC were stained with Giemsa (diluted 1:10 in distilled water) for 15 min. The number of ES, representing tube-like formation capability of HUVEC, was scored by two observers in 10 fields using a contrast phase microscope (Olympus CKX FA) with 10× magnification.

### Quantitative Real Time PCR (qRT-PCR)

Subconfluent cultures of LN18 and LN229 cells were placed in SFM for 24 and 48 h, and then RNA was isolated using Trizol reagent (Invitrogen), according to manufacturer's instructions. A total of 10 μg of RNA was reverse transcribed in 100 μl of reaction volume using the High-Capacity cDNA Archive (Applied Biosystems, Foster City, CA) according to the manufacturer's protocol. Seven μl of the RT products were used to amplify leptin and VEGF sequences using the Hs00174877_m1 and the Hs00900054_m1 TaqMan probes (Applied Biosystems), respectively. To normalize qRT-PCR reactions, parallel reactions were run on each sample for β-actin. Changes in the target mRNA content relative to β-actin mRNA were determined using a comparative CT method to calculate changes in CT, and ultimately fold and percent change. An average CT value for each RNA was obtained for replicate reactions.

### Western blot (WB) analysis

Subconfluent cultures (80% of density) of HUVEC were placed in SFM for 1 h, pretreated for 1 h with ObR or VEGFR inhibitors, and then treated with 200 ng/mL leptin or 50 ng/mL VEGF for 15 min or left untreated. Next, the cells were lysed in a buffer containing 1% NP40, 50 mM HEPES pH 7.5, 250 mM NaCl, 5 mM EDTA pH 8.0, 0.1% SDS, protease inhibitors 1× (Complete Mini EDTA-free protease inhibitors, Roche, Germany) and phosphatase inhibitors (10 mM Na_3_Vo_4 _and 50 mM NaF). The expression of proteins was analyzed in 50-70 μg of total cell lysates. The following antibodies (Ab) were used for WB: for phospho-STAT3, STAT3 Tyr705, D3A7 mAb, 1:1000 and for total STAT3, STAT3 79D7 mAb, 1:1000, all purchased from Cell Signaling, MA, USA; for glyceraldehyde-3-phosphate dehydrogenase 6C5 (GAPDH), 1:1000 (Santa Cruz Biotechnology, CA, USA).

### Leptin and VEGF detection by ELISA

CM obtained from 2-3 plates of 80% confluent GBM cultures was collected, as described above. The concentrations of leptin and VEGF in CM (obtained using WB lysis buffer without 0.1% SDS) were measured using leptin and VEGF Human Quantikine ELISA Kits (R&D Systems). The standard curve was created using purified leptin or VEGF. The concentrations of leptin or VEGF are expressed as pg/mL/9 × 10^6 ^LN18 cells and pg/mL/6 × 10^6 ^LN229 cells. All detected concentrations were within the range of the standard curve. All measurements were done in triplicate and the experiments were repeated three times.

### Statistical analysis

All experiments were done at least in triplicates and data analyzed by Student's t-test. Differences with p values of ≤ 0.05 were considered significant.

## Competing interests

The authors declare that they have no competing interests.

## Authors' contributions

RF carried out the majority of experiments, analyzed and elaborated data, and drafted the manuscript, MB carried out experiments, LO prepared the ObR antagonist and analyzed data, ES designed the study, analyzed data and edited the final manuscript. All authors read and approved the final manuscript.

## Pre-publication history

The pre-publication history for this paper can be accessed here:

http://www.biomedcentral.com/1471-2407/11/303/prepub

## References

[B1] Friedman JM Halaas JL Leptin and the regulation of body weight in mammals Nature 1998 395 6704 763 770 10.1038/27376 9796811

[B2] Zhang F Chen Y Heiman M Dimarchi R Leptin: structure, function and biology Vitam Horm 2005 71 345 372 10.1016/S0083-6729(05)71012-8 16112274

[B3] Cao Y Angiogenesis modulates adipogenesis and obesity J Clin Invest 2007 117 9 2362 2368 10.1172/JCI32239 PMC1963348 17786229

[B4] Rahmouni K Haynes WG Endothelial effects of leptin: implications in health and diseases Curr Diab Rep 2005 5 4 260 266 10.1007/s11892-005-0020-5 16033675

[B5] Vona-Davis L Rose DP Angiogenesis, adipokines and breast cancer Cytokine Growth Factor Rev 2009 20 3 193 201 10.1016/j.cytogfr.2009.05.007 19520599

[B6] Bouloumie A Drexler HC Lafontan M Busse R Leptin, the product of Ob gene, promotes angiogenesis Circ Res 1998 83 10 1059 1066 10.1161/01.res.83.10.1059 9815153

[B7] Sierra-Honigmann MR Nath AK Murakami C Garcia-Cardena G Papapetropoulos A Sessa WC Madge LA Schechner JS Schwabb MB Polverini PJ Flores-Riveros JR Biological action of leptin as an angiogenic factor Science 1998 281 5383 1683 1686 10.1126/science.281.5383.1683 9733517

[B8] Artwohl M Roden M Holzenbein T Freudenthaler A Waldhausl W Baumgartner-Parzer SM Modulation by leptin of proliferation and apoptosis in vascular endothelial cells Int J Obes Relat Metab Disord 2002 26 4 577 580 10.1038/sj.ijo.0801947 12075587

[B9] Park HY Kwon HM Lim HJ Hong BK Lee JY Park BE Jang Y Cho SY Kim HS Potential role of leptin in angiogenesis: leptin induces endothelial cell proliferation and expression of matrix metalloproteinases in vivo and in vitro Exp Mol Med 2001 33 2 95 102 10.1038/emm.2001.17 11460888

[B10] Anagnostoulis S Karayiannakis AJ Lambropoulou M Efthimiadou A Polychronidis A Simopoulos C Human leptin induces angiogenesis in vivo Cytokine 2008 42 3 353 357 10.1016/j.cyto.2008.03.009 18448353

[B11] Cao R Brakenhielm E Wahlestedt C Thyberg J Cao Y Leptin induces vascular permeability and synergistically stimulates angiogenesis with FGF-2 and VEGF Proc Natl Acad Sci USA 2001 98 11 6390 6395 10.1073/pnas.101564798 PMC33478 11344271

[B12] Rouet-Benzineb P Aparicio T Guilmeau S Pouzet C Descatoire V Buyse M Bado A Leptin counteracts sodium butyrate-induced apoptosis in human colon cancer HT-29 cells via NF-kappaB signaling J Biol Chem 2004 279 16 16495 16502 10.1074/jbc.M312999200 14752104

[B13] Russo VC Metaxas S Kobayashi K Harris M Werther GA Antiapoptotic effects of leptin in human neuroblastoma cells Endocrinology 2004 145 9 4103 4112 10.1210/en.2003-1767 15166121

[B14] Hoda MR Keely SJ Bertelsen LS Junger WG Dharmasena D Barrett KE Leptin acts as a mitogenic and antiapoptotic factor for colonic cancer cells Br J Surg 2007 94 3 346 354 10.1002/bjs.5530 17212381

[B15] Catalano S Giordano C Rizza P Gu G Barone I Bonofiglio D Giordano F Malivindi R Gaccione D Lanzino M De Amicis F Andò S Evidence that leptin through STAT and CREB signaling enhances cyclin D1 expression and promotes human endometrial cancer proliferation J Cell Physiol 2009 218 3 490 500 10.1002/jcp.21622 18988190

[B16] Saxena NK Vertino PM Anania FA Sharma D leptin-induced growth stimulation of breast cancer cells involves recruitment of histone acetyltransferases and mediator complex to CYCLIN D1 promoter via activation of Stat3 J Biol Chem 2007 282 18 13316 13325 10.1074/jbc.M609798200 PMC2923657 17344214

[B17] Okumura M Yamamoto M Sakuma H Kojima T Maruyama T Jamali M Cooper DR Yasuda K Leptin and high glucose stimulate cell proliferation in MCF-7 human breast cancer cells: reciprocal involvement of PKC-alpha and PPAR expression Biochim Biophys Acta 2002 1592 2 107 116 10.1016/s0167-4889(02)00276-8 12379472

[B18] Frankenberry KA Somasundar P McFadden DW Vona-Davis LC Leptin induces cell migration and the expression of growth factors in human prostate cancer cells Am J Surg 2004 188 5 560 565 10.1016/j.amjsurg.2004.07.031 15546570

[B19] Jaffe T Schwartz B Leptin promotes motility and invasiveness in human colon cancer cells by activating multiple signal-transduction pathways Int J Cancer 2008 123 11 2543 2556 10.1002/ijc.23821 18767036

[B20] Saxena NK Sharma D Ding X Lin S Marra F Merlin D Anania FA Concomitant activation of the JAK/STAT, PI3K/AKT, and ERK signaling is involved in leptin-mediated promotion of invasion and migration of hepatocellular carcinoma cells Cancer Res 2007 67 6 2497 2507 10.1158/0008-5472.CAN-06-3075 PMC2925446 17363567

[B21] Sharma D Saxena NK Vertino PM Anania FA Leptin promotes the proliferative response and invasiveness in human endometrial cancer cells by activating multiple signal-transduction pathways Endocr Relat Cancer 2006 13 2 629 640 10.1677/erc.1.01169 PMC2925427 16728588

[B22] Yeh WL Lu DY Lee MJ Fu WM Leptin induces migration and invasion of glioma cells through MMP-13 production Glia 2009 57 4 454 464 10.1002/glia.20773 18814267

[B23] Grosfeld A Andre J Hauguel-De Mouzon S Berra E Pouyssegur J Guerre-Millo M Hypoxia-inducible factor 1 transactivates the human leptin gene promoter J Biol Chem 2002 277 45 42953 42957 10.1074/jbc.M206775200 12215445

[B24] Cascio S Bartella V Auriemma A Johannes GJ Russo A Giordano A Surmacz E Mechanism of leptin expression in breast cancer cells: role of hypoxia-inducible factor-1alpha Oncogene 2008 27 4 540 547 10.1038/sj.onc.1210660 17653093

[B25] Koda M Sulkowska M Kanczuga-Koda L Cascio S Colucci G Russo A Surmacz E Sulkowski S Expression of the obesity hormone leptin and its receptor correlates with hypoxia-inducible factor-1 alpha in human colorectal cancer Ann Oncol 2007 18 Suppl 6 vi116 119 10.1093/annonc/mdm238 17591803

[B26] Koda M Sulkowska M Wincewicz A Kanczuga-Koda L Musiatowicz B Szymanska M Sulkowski S Expression of leptin, leptin receptor, and hypoxia-inducible factor 1 alpha in human endometrial cancer Ann N Y Acad Sci 2007 1095 90 98 10.1196/annals.1397.013 17404022

[B27] Bartella V Cascio S Fiorio E Auriemma A Russo A Surmacz E Insulin-dependent leptin expression in breast cancer cells Cancer Res 2008 68 12 4919 4927 10.1158/0008-5472.CAN-08-0642 18559540

[B28] Garofalo C Koda M Cascio S Sulkowska M Kanczuga-Koda L Golaszewska J Russo A Sulkowski S Surmacz E Increased expression of leptin and the leptin receptor as a marker of breast cancer progression: possible role of obesity-related stimuli Clin Cancer Res 2006 12 5 1447 1453 10.1158/1078-0432.CCR-05-1913 16533767

[B29] Carino C Olawaiye AB Cherfils S Serikawa T Lynch MP Rueda BR Gonzalez RR Leptin regulation of proangiogenic molecules in benign and cancerous endometrial cells Int J Cancer 2008 123 12 2782 2790 10.1002/ijc.23887 PMC2892183 18798554

[B30] Gonzalez RR Cherfils S Escobar M Yoo JH Carino C Styer AK Sullivan BT Sakamoto H Olawaiye A Serikawa T Leptin signaling promotes the growth of mammary tumors and increases the expression of vascular endothelial growth factor (VEGF) and its receptor type two (VEGF-R2) J Biol Chem 2006 281 36 26320 26328 10.1074/jbc.M601991200 16825198

[B31] Rene Gonzalez R Watters A Xu Y Singh UP Mann DR Rueda BR Penichet ML Leptin-signaling inhibition results in efficient anti-tumor activity in estrogen receptor positive or negative breast cancer Breast Cancer Res 2009 11 3 R36 10.1186/bcr2321 PMC2716504 19531256

[B32] Brown R Morash B Ur E Wilkinson M RNAi-mediated silencing of leptin gene expression increases cell death in C6 glioblastoma cells Brain Res Mol Brain Res 2005 139 2 357 360 10.1016/j.molbrainres.2005.05.009 15964097

[B33] Morash B Johnstone J Leopold C Li A Murphy P Ur E Wilkinson M The regulation of leptin gene expression in the C6 glioblastoma cell line Mol Cell Endocrinol 2000 165 1-2 97 105 10.1016/s0303-7207(00)00259-8 10940488

[B34] Knerr I Schuster S Nomikos P Buchfelder M Dotsch J Schoof E Fahlbusch R Rascher W Gene expression of adrenomedullin, leptin, their receptors and neuropeptide Y in hormone-secreting and non-functioning pituitary adenomas, meningiomas and malignant intracranial tumours in humans Neuropathol Appl Neurobiol 2001 27 3 215 222 10.1046/j.0305-1846.2001.00324.x 11489141

[B35] Riolfi M Ferla R Del Valle L Pina-Oviedo S Scolaro L Micciolo R Guidi M Terrasi M Cetto GL Surmacz E Leptin and its receptor are overexpressed in brain tumors and correlate with the degree of malignancy Brain Pathol 2009 20 2 481 489 10.1111/j.1750-3639.2009.00323.x PMC2864337 19775291

[B36] Morash B Li A Murphy PR Wilkinson M Ur E Leptin gene expression in the brain and pituitary gland Endocrinology 1999 140 12 5995 5998 10.1210/endo.140.12.7288 10579368

[B37] Otvos L Jr Kovalszky I Scolaro L Sztodola A Olah J Cassone M Knappe D Hoffmann R Lovas S Hatfield MP Beko G Zhang S Wade JD Surmacz E Peptide-based leptin receptor antagonists for cancer treatment and appetite regulation Biopolymers 2010 96 117 126 10.1002/bip.21377 20564005

[B38] Bergers G Benjamin LE Tumorigenesis and the angiogenic switch Nat Rev Cancer 2003 3 6 401 410 10.1038/nrc1093 12778130

[B39] Jain RK di Tomaso E Duda DG Loeffler JS Sorensen AG Batchelor TT Angiogenesis in brain tumours Nat Rev Neurosci 2007 8 8 610 622 10.1038/nrn2175 17643088

[B40] Tate MC Aghi MK Biology of angiogenesis and invasion in glioma Neurotherapeutics 2009 6 3 447 457 10.1016/j.nurt.2009.04.001 PMC5084181 19560735

[B41] Vailhe B Vittet D Feige JJ In vitro models of vasculogenesis and angiogenesis Lab Invest 2001 81 4 439 452 10.1038/labinvest.3780252 11304563

[B42] Feng T Chen Y Shi G Yu X Wan C A collagen based vitro model of angiogenesis designed for tissue-engineering meterial Appl Surface Science 2008 255 312 314

[B43] Cascio S Ferla R D'Andrea A Gerbino A Bazan V Surmacz E Russo A Expression of angiogenic regulators, VEGF and leptin, is regulated by the EGF/PI3K/STAT3 pathway in colorectal cancer cells J Cell Physiol 2009 221 1 189 194 10.1002/jcp.21843 19492417

[B44] Knizetova P Ehrmann J Hlobilkova A Vancova I Kalita O Kolar Z Bartek J Autocrine regulation of glioblastoma cell cycle progression, viability and radioresistance through the VEGF-VEGFR2 (KDR) interplay Cell Cycle 2008 7 16 2553 2561 10.4161/cc.7.16.6442 18719373

[B45] Liu TJ Koul D LaFortune T Tiao N Shen RJ Maira SM Garcia-Echevrria C Yung WK NVP-BEZ235, a novel dual phosphatidylinositol 3-kinase/mammalian target of rapamycin inhibitor, elicits multifaceted antitumor activities in human gliomas Mol Cancer Ther 2009 8 8 2204 2210 10.1158/1535-7163.MCT-09-0160 PMC2752877 19671762

[B46] Zhang R Banik NL Ray SK Differential sensitivity of human glioblastoma LN18 (PTEN-positive) and A172 (PTEN-negative) cells to Taxol for apoptosis Brain Res 2008 1239 216 225 10.1016/j.brainres.2008.08.075 PMC2783255 18804099

[B47] Funahashi Y Shawber CJ Vorontchikhina M Sharma A Outtz HH Kitajewski J Notch regulates the angiogenic response via induction of VEGFR-1 J Angiogenes Res 2 1 3 10.1186/2040-2384-2-3 PMC2828996 20298529

[B48] Zhang H He Y Dai S Xu Z Luo Y Wan T Luo D Jones D Tang S Chen H Sessa WC Min W AIP1 functions as an endogenous inhibitor of VEGFR2-mediated signaling and inflammatory angiogenesis in mice J Clin Invest 2008 118 12 3904 3916 10.1172/JCI36168 PMC2575835 19033661

[B49] Boguslawski G McGlynn PW Harvey KA Kovala AT SU1498, an inhibitor of vascular endothelial growth factor receptor 2, causes accumulation of phosphorylated ERK kinases and inhibits their activity in vivo and in vitro J Biol Chem 2004 279 7 5716 5724 10.1074/jbc.M308625200 14625306

[B50] Chen SH Murphy DA Lassoued W Thurston G Feldman MD Lee WM Activated STAT3 is a mediator and biomarker of VEGF endothelial activation Cancer Biol Ther 2008 7 12 1994 2003 10.4161/cbt.7.12.6967 PMC2932444 18981713

[B51] Brandes AA Tosoni A Franceschi E Reni M Gatta G Vecht C Glioblastoma in adults Crit Rev Oncol Hematol 2008 67 2 139 152 10.1016/j.critrevonc.2008.02.005 18394916

[B52] Carmeliet P Jain RK Angiogenesis in cancer and other diseases Nature 2000 407 6801 249 257 10.1038/35025220 11001068

[B53] Kleihues P Soylemezoglu F Schauble B Scheithauer BW Burger PC Histopathology, classification, and grading of gliomas Glia 1995 15 3 211 221 10.1002/glia.440150303 8586458

[B54] Buatti J Ryken TC Smith MC Sneed P Suh JH Mehta M Olson JJ Radiation therapy of pathologically confirmed newly diagnosed glioblastoma in adults J Neurooncol 2008 89 3 313 337 10.1007/s11060-008-9617-2 18712283

[B55] Ryken TC Frankel B Julien T Olson JJ Surgical management of newly diagnosed glioblastoma in adults: role of cytoreductive surgery J Neurooncol 2008 89 3 271 286 10.1007/s11060-008-9614-5 18712281

[B56] Stupp R Mason WP van den Bent MJ Weller M Fisher B Taphoorn MJ Belanger K Brandes AA Marosi C Bogdahn U Curschmann J Janzer RC Ludwin SK Gorlia T Allgeier A Lacombe D Cairncross JG Eisenhauer E Mirimanoff RO European Organisation for Research and Treatment of Cancer Brain Tumor and Radiotherapy Groups National Cancer Institute of Canada Clinical Trials Group Radiotherapy plus concomitant and adjuvant temozolomide for glioblastoma N Engl J Med 2005 352 10 987 996 10.1056/NEJMoa043330 15758009

[B57] Batchelor TT Sorensen AG di Tomaso E Zhang WT Duda DG Cohen KS Kozak KR Cahill DP Chen PJ Zhu M Ancukiewicz M Mrugala MM Plotkin S Drappatz J Louis DN Ivy P Scadden DT Benner T Loeffler JS Wen PY Jain RK AZD2171, a pan-VEGF receptor tyrosine kinase inhibitor, normalizes tumor vasculature and alleviates edema in glioblastoma patients Cancer Cell 2007 11 1 83 95 10.1016/j.ccr.2006.11.021 PMC2748664 17222792

[B58] Vredenburgh JJ Desjardins A Herndon JE Dowell JM Reardon DA Quinn JA Rich JN Sathornsumetee S Gururangan S Wagner M Bigner DD Friedman AH Friedman HS Phase II trial of bevacizumab and irinotecan in recurrent malignant glioma Clin Cancer Res 2007 13 4 1253 1259 10.1158/1078-0432.CCR-06-2309 17317837

[B59] Ogunwobi OO Beales IL The anti-apoptotic and growth stimulatory actions of leptin in human colon cancer cells involves activation of JNK mitogen activated protein kinase, JAK2 and PI3 kinase/Akt Int J Colorectal Dis 2007 22 4 401 409 10.1007/s00384-006-0181-y 16912864

[B60] Fiorio E Mercanti A Terrasi M Micciolo R Remo A Auriemma A Molino A Parolin V Di Stefano B Bonetti F Giordano A Cetto GL Surmacz E Leptin/HER2 crosstalk in breast cancer: in vitro study and preliminary in vivo analysis BMC Cancer 2008 8 305 10.1186/1471-2407-8-305 PMC2588622 18945363

[B61] Garofalo C Sisci D Surmacz E Leptin interferes with the effects of the antiestrogen ICI 182,780 in MCF-7 breast cancer cells Clin Cancer Res 2004 10 19 6466 6475 10.1158/1078-0432.CCR-04-0203 15475434

[B62] Attoub S Noe V Pirola L Bruyneel E Chastre E Mareel M Wymann MP Gespach C Leptin promotes invasiveness of kidney and colonic epithelial cells via phosphoinositide 3-kinase-, rho-, and rac-dependent signaling pathways FASEB J 2000 14 14 2329 2338 10.1096/fj.00-0162 11053255

[B63] Morrison CD Leptin signaling in brain: A link between nutrition and cognition? Biochim Biophys Acta 2009 1792 5 401 408 10.1016/j.bbadis.2008.12.004 PMC2670357 19130879

[B64] Steppan CM Swick AG A role for leptin in brain development Biochem Biophys Res Commun 1999 256 3 600 602 10.1006/bbrc.1999.0382 10080944

[B65] Chen Z Htay A Dos Santos W Gillies GT Fillmore HL Sholley MM Broaddus WC In vitro angiogenesis by human umbilical vein endothelial cells (HUVEC) induced by three-dimensional co-culture with glioblastoma cells J Neurooncol 2009 92 2 121 128 10.1007/s11060-008-9742-y 19039523

[B66] Khodarev NN Yu J Labay E Darga T Brown CK Mauceri HJ Yassari R Gupta N Weichselbaum RR Tumour-endothelium interactions in co-culture: coordinated changes of gene expression profiles and phenotypic properties of endothelial cells J Cell Sci 2003 116 (Pt 6):1013 1022 10.1242/jcs.00281 12584245

[B67] Yao XH Ping YF Chen JH Chen DL Xu CP Zheng J Wang JM Bian XW Production of angiogenic factors by human glioblastoma cells following activation of the G-protein coupled formylpeptide receptor FPR J Neurooncol 2008 86 1 47 53 10.1007/s11060-007-9443-y 17611713

[B68] Otvos L Jr Kovalszky I Riolfi M Ferla R Olah J Sztodola A Nama K Molino A Piubello Q Wade JD Surmacz E Efficacy of a leptin receptor antagonist peptide in a mouse model of triple-negative breast cancer Eur J Cancer 2011 10.1016/j.ejca.2011.01.018 21353530

[B69] Otvos L Shao W-H Vanniashighe A Amon M Holub MC Kovalszky I Wade JD Doll M Cohen P Manolis N Surmacz E Towards understanding the role of leptin and leptin receptor antagonism in preclinical models of rheumatoid arthritis Biopolymers Peptide Science 2011 in press 10.1016/j.peptides.2011.06.015 21723351

[B70] Scolaro L Cassone M Kolaczynski JW Otvos L Jr Surmacz E Leptin-based therapeutics Expert Rev Endocrinol Metab 2010 5 875 889 10.1586/eem.10.61 30780830

[B71] Otvos L Jr Terrasi M Cascio S Cassone M Abbadessa G De Pascali F Scolaro L Knappe D Stawikowski M Cudic P Wade JD Hoffmann R Surmacz E Development of a pharmacologically improved peptide agonist of the leptin receptor Biochim Biophys Acta 2008 1783 10 1745 1754 10.1016/j.bbamcr.2008.05.007 18555805

[B72] Otvos L Jr Cassone M Terrasi M Cascio S Mateo GD Knappe D Hoffmann R Cudic P Wade JD Surmacz E Agonists and partial antagonists acting on the leptin--leptin receptor interface Adv Exp Med Biol 2009 611 497 498 10.1007/978-0-387-73657-0_215 19400282

